# Neutrophils: Musketeers against immunotherapy

**DOI:** 10.3389/fonc.2022.975981

**Published:** 2022-08-25

**Authors:** Kashif Rafiq Zahid, Umar Raza, Soumya Tumbath, Lingxiang Jiang, Wenjuan Xu, Xiumei Huang

**Affiliations:** ^1^ Department of Radiation Oncology, Melvin and Bren Simon Comprehensive Cancer Center, Indiana University School of Medicine, Indianapolis, IN, United States; ^2^ Department of Biological Sciences, National University of Medical Sciences (NUMS), Rawalpindi, Pakistan

**Keywords:** neutrophils, immunotherapy, immune checkpoints, NETs, EMT

## Abstract

Neutrophils, the most copious leukocytes in human blood, play a critical role in tumorigenesis, cancer progression, and immune suppression. Recently, neutrophils have attracted the attention of researchers, immunologists, and oncologists because of their potential role in orchestrating immune evasion in human diseases including cancer, which has led to a hot debate redefining the contribution of neutrophils in tumor progression and immunity. To make this debate fruitful, this review seeks to provide a recent update about the contribution of neutrophils in immune suppression and tumor progression. Here, we first described the molecular pathways through which neutrophils aid in cancer progression and orchestrate immune suppression/evasion. Later, we summarized the underlying molecular mechanisms of neutrophil-mediated therapy resistance and highlighted various approaches through which neutrophil antagonism may heighten the efficacy of the immune checkpoint blockade therapy. Finally, we have highlighted several unsolved questions and hope that answering these questions will provide a new avenue toward immunotherapy revolution.

## 1 Introduction

Neutrophils are the most profuse leukocytes, representing 50%–70% of all the circulating leukocytes in human, and are regarded as body’s first responders to injury, infections, and inflammation (1, 2). In response to infection-associated signals, neutrophils initiate multiple effector functions such as the production of neutrophil extracellular traps (NETs), generation of reactive oxygen species (ROS), and production of antibacterial peptides to eradicate pathogens (3, 4). The functional importance of neutrophils had been overlooked previously on the basis of their reported short life span; however, recent studies suggesting that they can survive in the circulation from 19 h to 5 days have ensured renewed attention toward the role of neutrophils under varying biological conditions (5). Since the last two decades, various immunotherapeutic agents have been approved as treatment for multiple human cancers, and most of them mainly focus on the targeting of major immunosuppressive molecules in both tumor and immune cells. Immune checkpoint blockade therapy has been extensively tested and approved as first-line treatment for various cancers (6, 7). Although the development of novel immune checkpoint inhibitors has emerged as a revolutionary milestone in defeating human tumors, and tuning immune system activity for promoting its antitumor activity and overcoming immune suppression, work from multiple groups has shown that immunotherapy treatments have largely failed in most of the patients with solid tumors (8–10). Undoubtedly, several combinatorial treatment approaches have improved these metrics. For instance, anti–programmed death-1 (anti-PD1) monoclonal antibody, pembrolizumab, combined with chemotherapy, has been proven effective in Non-small cell lung cancer (NSCLC) patients whereas treatment with another (anti-PD1) monoclonal antibody, nivolumab, combined with the monoclonal antibody ipilimumab, which enhances the T-cell response by targeting cytotoxic T lymphocyte–associated antigen 4 (CTLA-4), has been fruitful to treat cancer in advanced melanoma patients (11, 12). However, most of the cancer patients are still not getting satisfactory benefits from immune checkpoint blockade therapy due to either a low response rate or higher immune-related toxicities (13). Recently, it has been reported that nearly 44% of the US population of tumor patients is eligible for checkpoint blockade therapy and merely approximately 13% of the patients showed a positive response to it (14). This adverse outlook is associated with multidimensional tumor microenvironment (TME), which endlessly formulates unique resistance mechanisms, thereby leading to a limited response to immunotherapeutic agents (15). Neutrophils are emerging as central effector cells of the innate immune system and are associated with poor outcomes in many types of human cancers, except some specific tumor types (16, 17). Accumulating evidence suggests that neutrophils are key components of TME, drive tumor progression, and limit the efficacy of immunotherapy by establishing immunosuppressive TME (18–20). Furthermore, neutrophils also counteract immunotherapy efficacy by manipulating the adaptive immune system (21, 22). In recent years, boosting the antitumor ability of immune cells, particularly of neutrophils in the tumor niche, has become a major goal in devising new treatment options, owing to aggressive immunosuppressive TME. That is why we have focused on the relationship between neutrophils in tumor progression and immune suppression in this review. We begin with unmasking the molecular pathways by which neutrophils are polarized into the antitumor (N1) or protumor (N2) phenotype and support tumor progression/suppression. Later, we discuss the key concepts related to the critical role of neutrophils in immune suppression/evasion and therapy resistance and highlight the novel strategies for targeting immunosuppressive neutrophils.

## 2 Neutrophils in cancer progression

**Figure 1:**
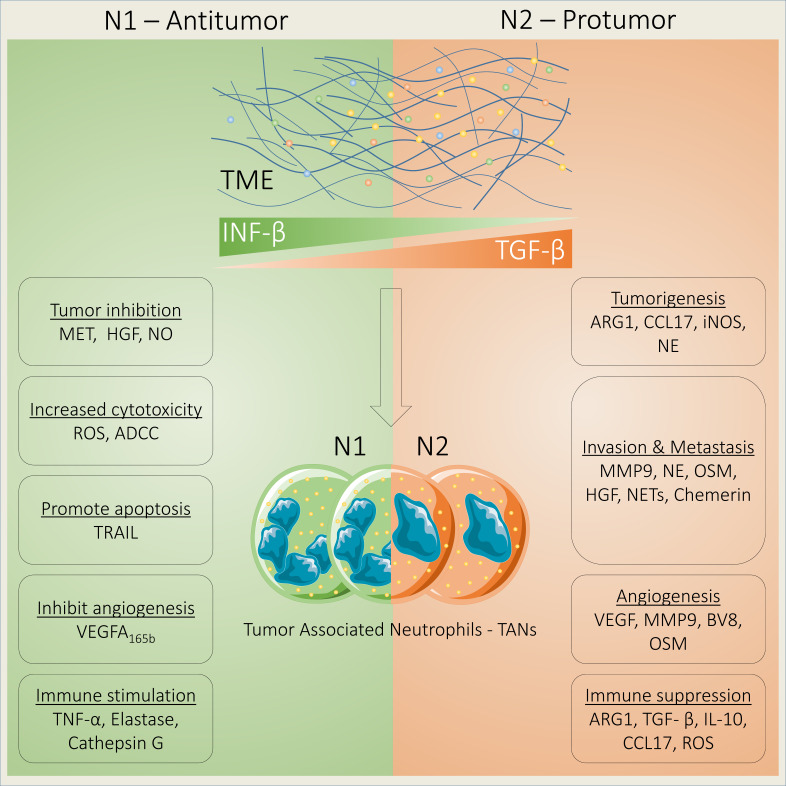
Neutrophils act as tumor suppressor or tumor promoter. Neutrophils act as either a tumor suppressor or promoter depending on their phenotypes that are regulated by transforming growth factor beta (TGF-β) and interferon beta (IFN-β). N1 TANs suppress tumor growth by increasing the expression of MET and HGF binding and NO production. In addition, N1 TANs increase cell toxicity *via* ADCC and ROS generation and apoptosis by releasing TRAIL; inhibit angiogenesis by releasing antiangiogenic VEGF-A_165b_; and induce immune stimulation by releasing TNF-α, elastase, and cathepsin G. In contrast, N2 TANs promote tumorigenesis through ARG1, CCL17, iNOS, and NE; foster invasion and metastasis *via* MMP9, NE, OSM, HGF, NETs, and chemerin; support angiogenesis by releasing VEGF, MMP9, BV8, and OSM; and induce immune suppression through ARG1, TGF-β, IL-10, CCL17, and ROS.

Since the last decade, the customary standpoint about neutrophils as a mere bystander in human tumors has been revolutionized and research on the diverse role of neutrophils in cancer progression was established significantly, which can be reflected by numerous recent review papers published in well-reputed journals (23, 24). Increased neutrophil abundance is frequently detected in both cancer patients and tumor-bearing mice (25, 26). A growing body of evidence also suggests that neutrophils function as early responders against inflammatory insult (27, 28). Recently, the neutrophil-to-lymphocyte ratio (NLR) has been used to predict a patient’s tumor or inflammatory status and immunotherapy response in multiple cancer types (29, 30). Neutrophils are capable to infiltrate into tumors and constitute a major portion of the TME (31). Furthermore, association between these tumor-associated neutrophils (TANs) and patient outcomes have also been demonstrated (32). Neutrophils act as a double-edged sword in human cancer, owing to their inimitable potential to either support or inhibit tumor progression. Strong evidence suggests that neutrophils act as a tumor promoter (33–36), while fewer studies have reported that neutrophils may also act as a tumor suppressor (37, 38). Moreover, both the preclinical and clinical trials have shown that TANs contribute in malignant transformation, angiogenesis, and antitumor immunity (39, 40). Based on their role in tumor progression, TANs can be divided into N1 and N2 types. The N1 TANs inhibit tumor growth and increase antitumor immune memory and tumor cell toxicity, while N2 TANs foster tumorigenesis, invasion, metastasis, and immune suppression (41). Tumor cell–driven TME factors often signal for TAN polarization into N1 and N2 types. For instance, transforming growth factor beta (TGF-β), the major immunosuppressive cytokine that is also correlated with poor prognosis in cancer patients, is released by tumor cells in the TME where it polarizes neutrophils to the N2 phenotype and suppresses N1-type neutrophils. On the other hand, interferon beta (IFN-β) in the TME suppresses the N2 neutrophil phenotype and stimulates N1 neutrophils (42, 43) (**Figure 1**). Changes in the expression of heat-shock proteins (HSPs) have been suggested as danger-associated molecular patterns (DAMPs) because these molecules are highly conserved and their intracellular expression is elevated in response to infection and oxidative stress (44). HSP72 as an endogenous DAMP activates neutrophils *via* TLR4 signaling (45). A previous study investigated the key role of TLR4 in the programming of N1/N2 neutrophils after stroke. Results showed that the absence of TLR4 increased the amount of N2 neutrophils in ischemic brain (46). Similarly, a recent study has shown that TLR4 regulates neutrophils dynamics in stroke (47). Together, the above findings suggest that HSPs as a danger signal may regulate neutrophil polarization. Here, we have reviewed the tumor- promoting role of neutrophils by focusing on N2-type TANs that exploit diverse mechanisms to promote tumor progression, such as secreting inflammatory cytokines and chemokines (48, 49), releasing NETs (50), and epithelial-to-mesenchymal transition (EMT) (51) (**Table 1**), which we have discussed in detail in the following subsections.

**Table 1:** Role of neutrophils in tumor progression.

Mediators	Interaction with neutrophils	Mechanism	References
**XIAP**	Increases neutrophil infiltration	XIAP-mediated IL-8 secretion promotes neutrophil recruitment and contributes to melanoma tumor progression	(20)
**TGF-β**	Promotes neutrophil recruitment	TGF-β-mediated neutrophils recruitment promotes colorectal tumor metastasis by activating NOTCH1	(52)
**CXCL5**	Increases neutrophil accumulation in lungs	CXCL5-mediated neutrophil accumulation in lungs promotes lung cancer metastasis by inhibiting the differentiation of antitumor CD8^+^ T cells	(53)
**CXCR2**	Increases neutrophil recruitment	CXCR2-mediated neutrophil recruitment contributes to brain metastasis through triggering NET formation	(54)
**IL-17**	Promotes neutrophil recruitment	IL-17-mediated neutrophil recruitment contributes to pancreatic tumor through inducing NET formation and inactivates antitumor CD8^+^ T cells	(55)
**IL-23/IL-18**	Promote neutrophils to secrete IL-17A and IL-22	IL-23 plus IL-18-mediated polarization of neutrophils to the Th17-type phenotype promotes lymphoma tumor through the JNK/P38-STAT3 axis	(56)
**GM-CSF**	Induces tumor-infiltrating PD-L1+ neutrophils	GM-CSF-induced tumor-infiltrating PD-L1^+^ neutrophils contributes to laryngeal squamous cell carcinoma by inhibiting T-cell proliferation and activation	(57)
**IL-8**	Induced by neutrophil receprtors CXCR1/2	IL-8/PTEN/STAT3/snail positive feedback loop induces EMT, thereby leading to head and neck squamous cell carcinoma proliferation and metastasis	(58)
**C3aR**	Expressed by neutrophils	Neutrophil-mediated induction of C3aR triggers NETosis and coagulation, polarizing neutrophils to the N2 phenotype and promotes intestinal tumorigenesis	(59)
**TIMP-1**	Released by neutrophils	Neutrophil-mediated production of TIMP-1 induces EMT and contributes to breast cancer metastasis	(60)
**MMP8**	Expressed by neutrophils	Reciprocal positive interaction between MMP-8 and TGF**-**β1 promotes hepatocellular carcinoma progression by inducing EMT *via* PI3K/Akt/Rac1 axis	(61)
**NE**	Secreted by neutrophils	Neutrophil-secreted elastase contributes to oral tumor cell survival and invasion through Src/PI3K-dependent activation of the AKT signaling	(36)

### 2.1 Inflammatory cytokines and chemokines

Tumor cells induce various inflammatory cytokines, including TNF-α, IL-1β, IL-6, IL-17/18, and IL-23, and growth factors (G-CSF, GM-CSF, and IL-3) to generate neutrophil production and confirm their survival (55–57, 62). Furthermore, various neutrophil-attracting chemokines (CXCL1, CXCL2, CXCL5, CXCL6, and CXCL8) promote the migration of neutrophils to the tumor site through CXCR1 and CXCR2 receptors (54, 63). More recent study has shown that the cancer cell-mediated secretion of CXCL5 drives mature protumorigenic neutrophil infiltration in non-small cell lung cancer and impairs the differentiation of antitumor CD8^+^ T cells (53). CXCL8 is highly expressed in a wide range of tumor types, and various studies have suggested that the CXCL8 serum level in tumor patients serves as an independent prognostic marker (64, 65). Moreover, it supports tumor progression and promotes resistance to immune checkpoint blockade therapy (65, 66). The function of CXCL8 mainly depends on its binding with two receptors, namely, CXCR1 and CXCR2 (67). Notably, these two receptors are highly expressed on neutrophils (68, 69). The inhibition of CXCR1/2 limits neutrophil infiltration and results in the decreased growth of multiple tumors, including lung adenocarcinoma (70), colorectal cancer (71), and pancreatic ductal adenocarcinoma (72). Furthermore, inflammatory cytokines foster the production of growth factor G-CSF in the TME and further stimulate new neutrophil production in bone marrow (73, 74). In turn, neutrophils stimulate tumor-associated inflammation, thereby leading to tumor progression. In this line, more recently, Shan et al. have investigated interaction among neutrophils, CD4^+^ T cells, and tumor cells in the gastric TME. Their findings show that CXCL6/CXCL8-CXCR1 chemotaxis arbitrates neutrophil recruitment and accumulation into the gastric TME, an event which upregulates the expression of CD54 and B7-H2 through the activation of the Extracellular signal-regulated kinase (ERK), Nuclear factor kappa B (NF-κB) pathway by tumor-derived TNF-α. Then, neutrophils induce the polarization of the IL-17A-generating Th subsets in a B7-H2-dependent manner, where polarized IL-17A-generating Th cells can be able to wield protumorigenic roles through IL-17A, thereby leading to gastric tumor onset and progression (75). In pancreatic cancer, the expression of neutrophil chemoattractants in tumor cells is increased, following gemcitabine treatment. Subsequently, Gas6-expressing neutrophils infiltrate and accumulate in liver in a CXCR-2-dependent manner. Then, neutrophil-derived Gas6 induces AXL Receptor Tyrosine Kinase (AXL) on metastatic tumor cells and finally contributes to metastatic growth in liver. Furthermore, the pharmacological targeting of the Gas6/AXL axis through warfarin in combination with gemcitabine treatment suppresses metastatic relapse (76). Another latest study has revealed that IL-17-mediated neutrophil infiltration contributes to gastric tumor angiogenesis and maintains tumor persistence (77). These novel findings highlight the crucial role of cytokines and chemokines in neutrophil infiltration to trigger tumor onset and progression and the impact of their blocking to improve efficacy of immunotherapy. Moreover, a deep knowledge about their function in neutrophil maturation and activation can further guide to establish more effective therapeutic strategies.

### 2.2 Neutrophil extracellular traps

Another important mechanism by which neutrophils promote tumor progression is NETs. NETs are web-like DNA structures and contain the abundance of antimicrobial proteins that are released by neutrophils through a unique program cell death process termed NETosis induced by different pro-inflammatory mediators and microbial stimuli (78, 79). The major NETosis inducers in tumor can vary between different tumor models, but HMGB1 (80) and CXCR1/2 (81, 82) agonists have been found to induce tumor-associated NETosis. Recently, the role of NETs is conspicuously becoming critical in promoting tumor progression (83, 84). Previously, various studies have identified the abundance of NETs in mouse tumor models (59, 85, 86). Recently, the abundance of NETs has been detected and quantified in many types of solid tumors (87) and can be used as tumor biomarker candidates for clinical diagnosis (88, 89). NETs stimulate tumor growth (90) and serve as a scaffold for the inert adhesion or chemotaxis of the cancer cells in different tumor types such as breast, liver, and colon cancer (91, 92). NETs are stimulated by pro-inflammatory cytokines or chemokines during infection (93, 94). And promote tumor progression through diverse mechanisms such as by awakening dormant tumor cells in lungs (95), inducing tumor cell chemotaxis to the liver (84), modulating cancer cell bioenergetics (96), and building a protective coat around cancer for protecting them against drug cytotoxicity (55).

Tumor metastasis is the major reason of huge number of cancer-associated deaths (97). In recent years, emerging studies have shown that NETs foster tumor metastasis in a wide range of cancer types (98–100), whereas NET depletion markedly decreases tumor metastasis (101). In line with these reports, in a more recent study published in Cancer Cell, Xiao et al. have identified a novel mechanism by indicating that NETs promote lung metastasis by degrading TSP-1 protein. In addition, this novel research has revealed a new pathway by which CTSC expression promotes metastatic potential through activating neutrophil membrane–bound PR3 to assist in IL-1β processing and the activation of NF-κB, thereby leading to the upregulation of IL-6 and CCL3 to recruit neutrophils in the metastatic lung niche, while the targeting of CTSC by AZD7986 avoids mouse lung metastasis (92). IL-17 has also been found to promote pancreatic ductal adenocarcinoma progression and immune checkpoint blockade therapy resistance by triggering NET formation, while IL-17 blockade enhances immune checkpoint blockade sensitivity (55). Similarly, another recent study has shown that NETs trap HCC cells and fuel their metastatic potential by activating the TLR4/9-COX2 axis, whereas the inhibition of COX2 by using HCQ and TLR4/9 by Dnase I effectively suppresses HCC metastasis. This combinatorial approach not only efficiently depletes NETs but also abrogates the metastatic ability of the trapped liver cancer cells *via* undissolved NETs (91).

### 2.3 Epithelial-to-mesenchymal transition

EMT is a phenotypic switching event where epithelial cells lose their characteristics and undergo mesenchymal transition. The EMT event is orchestrated by the activation of various transcription factors (TFs) such as TWIST1/2, ZEB1/2, and SNAIL1/2 (102), growth factors (TGF-β and Hepatocyte growth factor (HGF), and inflammatory cytokines (TNF-α, CXCL12, IL-6, and IL-8), which, in turn, drive cancer cell invasion (103, 104). Interestingly, same EMT-TFs regulate the expression of various secreted mediators such as growth factors (GM-CSF), cytokine (TNF-α), and chemokines (CXCL6/8/11, CCL2, and GRO) in cancer cells (105–107). In addition, numerous mediators from EMT-induced tumors produce monocytes (CCL2) and neutrophil chemoattractants (GM-CSF, CXCL8, and GRO); the production of these mediators following the induction of EMT regulate the tumor niche immune landscape (108). It has also been reported that neutrophils interact with tumor cells by using the same molecular machinery that induces EMT such as TGF-β, IL-8, CCl2, TNF-α, and IL-17a (61, 100). Consistently, TGF-β pathways stimulate both EMT and the neutrophil pro-tumor phenotype (N2), which is likewise associated with therapy resistance (101). Emerging evidence suggests that neutrophils stimulate tumor progression through inducing EMT (60, 109, 110). It is well documented that both the neutrophils and EMT play a critical role in tumor progression and immunotherapy resistance (105, 111). However, the underlying molecular mechanism of neutrophil-mediated EMT induction remains poorly understood. Here, we provide a recent update about how neutrophils foster tumor through inducing EMT. In gastric cancer stroma, neutrophils promote tumor metastasis by inducing EMT through secreting CXCL5 and IL-17a, whereas antibody-mediated IL-17a blockade suppresses EMT in cancer cells cocultured with TANs (112, 113). In breast tumor, neutrophils induce EMT by producing TIMP-1. TANs-MCF-7 interaction establishes a feedback-loop between MCF-7 cells and TANs with the induction of EMT in cancer cells due to the increased expression of TIMP-1 by CD90. Blocking CD90 decreases tumor metastasis in mice (114). Authors have identified the novel mechanism of the neutrophil-mediated induction of EMT in breast cancer cells by regulating TIMP-1. However, the exact molecular mechanism of how CD90 regulates TIMP-1 expression in neutrophils remains unexplored. In addition, this novel study also provides the foundation to further investigate the therapeutic effect of CD90 blockade in different tumor types.

## 3 Neutrophils as immune suppressor

**Figure 2:**
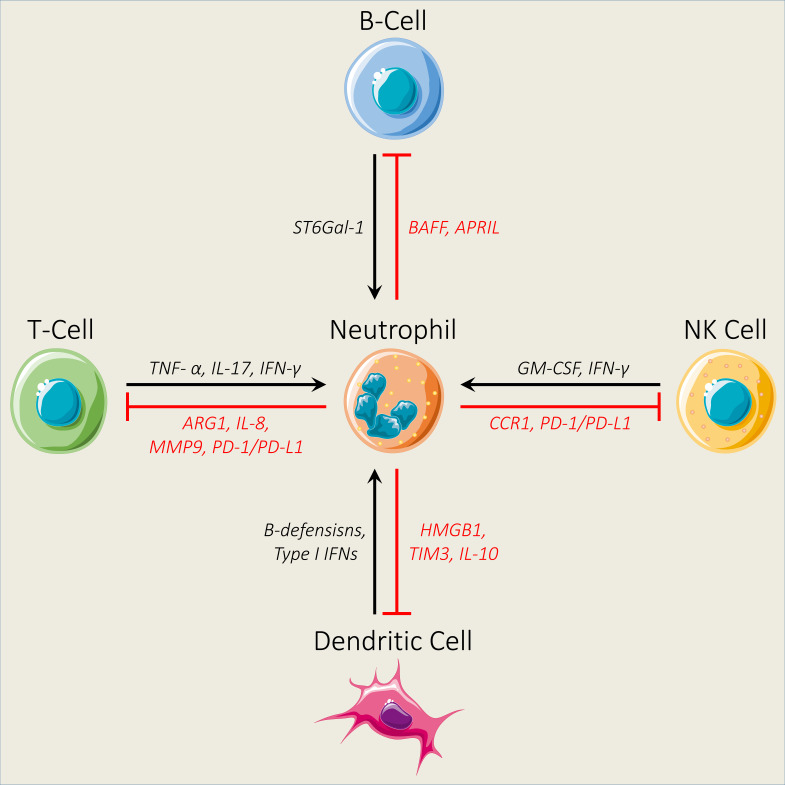
Interaction between neutrophils and different immune cells. Neutrophils impair the infiltration ability of natural killer (NK) cells by decreasing the expression levels of CCR1. In addition, neutrophils inhibit the antitumor activity of NK cells through the PD-1/PD-L1 axis. NK cells regulate neutrophil activation and survival by secreting GM-CSF and IFN-γ. Neutrophils modulate B-cell activation by secreting BAFF and APRIL, while B cells regulate neutrophil production through ST6Gal-1. Furthermore, neutrophils inhibit T-cell activity by increasing the expression levels of ARG1, IL-8, MMP9, and PD-1/PD-L1, while TNF- α, IL-17, and IFN-γ secreted by T cells promote neutrophil activation and recruitment. Finally, neutrophils N2 and dendritic cell interaction *via* the release of HMGB1 from neutrophils and TIM3 from dendritic cells and the production of IL-10 contribute to dendritic cell inhibition, while dendritic cells regulate neutrophil proliferation and survival by releasing β-defensins and Type 1 IFNs.

Many tumor therapeutic approaches, including radiotherapy, chemotherapy, and immunotherapy, have been widely used to control the level of neutrophil infiltrations or modulate their accumulation and function (25). However, neutrophils are linked with a poor clinical response to these targeted tumor therapies and mediate therapeutic resistance in most of the solid tumors because of their immunosuppressive features (115–117). Accumulating evidence suggests that neutrophils instigate the immunosuppressive TME through instituting brawny crosstalk both *in vitro* and *in vivo* with innate immune cells, including natural killer (NK) cells and dendritic cells (DCs), and adaptive immune cells including T cells and B cells (25, 118) (**Figure 2**). Therefore, it is critical to unmask the involved mechanisms for a better understanding of the crosstalk mediators and signaling pathways that dictate neutrophil infiltration into tumors, which can assist to design new effective therapeutic approaches. In this section, we will discuss the interaction between neutrophils and immune cells and how this crosstalk contributes to neutrophil-mediated immune suppression.

### 3.1 Interaction between neutrophils and T cells

T cells play a major role in effective antitumor immunity and are critical for tumor immunotherapy (119, 120). Recently, various studies have demonstrated the importance of the tumor-specific T-cell priming and activation in the draining lymph node, migration of the T cells toward the tumor site, and the creation of tertiary lymphoid structures inside tumor (112, 121). Primed T cells in the tumor-draining lymph node can respond more efficiently to immune checkpoint blockade compared with those T cells that enter and reside inside tumor (60, 117, 122). However, neutrophils contribute to the immunosuppressive microenvironment by suppressing antitumor T cells (123, 124). Although many scientists have investigated the role of neutrophils in tumor, how neutrophils and T cells communicate with each other to institute immune suppression remains elusive. Emerging evidence has suggested that the suppression of T-cell activity by neutrophils is mediated through the overexpression of ARG1 (125, 126) or the PD-1/PD-L1 axis (127, 128). Recent evidence suggests that TANs are associated with a poor clinical prognosis and also contribute to immune suppression by suppressing the activity of CD8^+^ T cells. In this event, IL-8 has been identified as a key player that establishes immunosuppressive crosstalk between TANs and CD8^+^ T cells through recruiting neutrophils into the TME and by inducing JAG2 (129). Another recent study has investigated the underlying molecular mechanism of the neutrophil-mediated inhibition of T-cell activity. Their novel findings demonstrate that neutrophils inhibit antitumor T-cell activity *via* the MMP-mediated induction of active TGF-β within the colon TME. Importantly, the depletion of neutrophils in mouse adenomas has resulted in decreased tumor burden and a high level of tumor-infiltrating T cells, while T-cell depletion, in turn, enhanced tumor burden and also abolished the valuable effects of neutrophil depletion. Together, these findings demonstrate that neutrophils drive colon tumor progression in mice by repressing the antitumor activity of T cells (130). Interestingly, Minns et al. have recently identified that primed and resting neutrophils contribute to opposite responses in T cells. Their findings show that resting neutrophils inhibit the activation of early-activating CD4^+^ and CD8^+^ T cells, while primed neutrophils do not inhibit activation significantly. Furthermore, neutrophils primed with unlike mediators show opposite effects on T cells. Neutrophils primed with LPS and TNF enhance CD4^+^ T-cell activation, while cytochalasin B/fMLF prime cells repress the activation of the late-stage T cells (131). Accumulating evidence suggests that neutrophils modulate the function of various T-cell subsets Th1, Th2, Th17, *γδ T*, and Treg cells (132–134). The interaction between TANs and T-cell responses has demonstrated the evidence of direct TAN-mediated inhibition of the Th1 cells and cytotoxic T lymphocytes in tumors. In this perspective, ARG1-expressing human granulocytic cells contribute to the downregulation of the CD3ζ chain on T cells *via* the depletion of L-arginine and ultimately suppress cytokine secretion and T-cell proliferation. In non-small cell lung cancer patients, ARG1^+^ neutrophils are increased with the disease stage in treatment-naïve patients and negatively correlated with the CD8^+^ T-cell population (135). Th17 cells produce different types of cytokines such as GM-CSF, TNF-α, IL-17, and IL-22 and acts as either antitumor or protumor (136, 137). Neutrophils secrete chemokines and cytokines that directly affect Th17 activation and differentiation (138, 139). A more recent study has shown that NETs regulate Th17 cell differentiation and activity through their histone protein components. This modulation of neutrophils, NETs, and histone protein components is mediated through TLR2 in T cells, thereby resulting in STAT3 phosphorylation (3). Coffelt et al. have investigated the critical role of IL-17-secreting cells and cancer-mediated inflammation in a metastasis event. Their findings show that IL-1 *β*-mediated-IL-17 induction from *γδ* T cells results in neutrophil expansion and polarization *via* G-CSF in mammary tumor-bearing mice, while the absence of neutrophils or *γδ* T cells significantly decreases tumor metastasis (140). These findings demonstrate a novel urbane neutrophil–T-cell interaction and highlight the critical need of exploring a new mediator of this interaction to induce T-cell-mediated adaptive immunity.

### 3.2 Interaction between neutrophils and B cells

Previously, immune checkpoint blockade therapy mainly focused on the bolstering of effector T lymphocytes; however, work from multiple laboratories have unmasked the fact that B cells are also key players of immunotherapy (141, 142). B cells possess both the protumor and antitumor functions, which mainly depend on their immune-suppressive or immune-stimulatory events and also the tumor type (143, 144). Reflective of both the protumor and antitumor activities of B cells, several clinical trials have been conducted to modulate B-cell functions (145). A recent study has shown that the adoptive transfer of the cancer-specific memory B cells display effective tumor suppression in a murine tumor model, thereby suggesting their clinical worth (146). Furthermore, B cells have also been reported to modulate the functional features of T cells (147, 148). In addition, the existence of PD-1^+^ and PD-L1^+^ B cells has been found in several types of human tumors (149–151). The baseline density of B cells can predict a response to immune checkpoint blockade therapy in tumor patients (152). Accumulating evidence suggests that neutrophils directly modulate the response of B cells by regulating cytokine production required for B-cell maturation, differentiation, and survival, such as BAFF and APRIL (153–155). Regarding human cancer, there are supporting evidences about the key role of neutrophils in the differentiation of the B cells. For instance, neutrophils are implicated in the pathogenesis of the B-cell lymphomas by producing APRIL (156). Moreover, it has been found that CXCL8 acts as a mediator to recruit APRIL-expressing neutrophils for diffusing B-cell lymphoma lesions (157). Another important mechanism by which neutrophils promote the development of B-cell chronic lymphocytic leukemia in mice is by elevating the expression level of APRIL and BAFF (158). Furthermore, the critical role of NETs and their crosstalk with CD5+ B cells is another important pathological mechanism driving B-cell chronic lymphocytic leukemia in mice (159). In a recent study in Nature, Petitprez et al. identified that patients with sarcoma immune class highly responded to immune checkpoint inhibitors, while patients from the immune class desert showed no response (160). These results have provided novel insights for changing the care of the patients with soft tissue sarcoma who showed a poor response to immune checkpoint blockade. In melanoma, B-cell signature, and not the T-cell signature, was associated with a response to immune checkpoint blockade. In addition, the density of B cells was enhanced in the tumor of responders compared to non-responding patients (152). B cells have been reported to penetrate into tumor and regulate tumor immunity. However, the profound impact of neutrophils on B cells has been overlooked. A recent study has shown that tumor-associated neutrophils (TANs) drive B-cell recruitment and modulation into plasma cells in the TME. In this event, TNF-α has been found as a key cytokine mediating B-cell chemotaxis by TANs (161). Recently, IL-10^+^ plasmablasts and IL-10^+^ B cells have been recovered from tumor-draining lymph nodes and tumor, where tumor patients have demonstrated a higher level of IL-10^+^ Bregs (162, 163). In addition, the key role of Bregs in regulating CD8^+^ T-cell responses has been reported in various tumor experimental models (164, 165). Bregs regulate immunity through various anti-inflammatory cytokines, including IL-10 and IL-35 (166). Moreover, Bregs inhibit CD4^+^ T-cell proliferation and Th1/17 differentiation through IL-10 and IL-35 (167). Accumulating evidence has suggested that neutrophils express IL-10 during inflammatory conditions (168); hence, neutrophils may modulate Breg-mediated immunity regulation by secreting IL-10.

### 3.3 Interaction between neutrophils and dendritic cells

Dendritic cells (DCs) are the most potent antigen-presenting cells (APCs) and play a critical role in innate and adaptive immune response *via* arresting, processing and presenting antigens to B and T cells and by activating antitumor T cells (169, 170). The antitumor response mainly depends on APCs to prime naïve T cells (171). Various studies have reported that the cDC1 subset is associated with the induction of tumor-controlling immunity and improved overall survival in many tumor types (172, 173). The activation of neutrophils releases the granule content neutrophils elastase (NE) that induces the polarization of the DC-mediated development of T cells into Th 17 cells, while the blocking of neutrophil activation or NE prevents the production of Th 17 cells (174). Another study has shown that neutrophils promote the development of Th 17 cells from naïve T cells preferentially through their interaction with DCs (175). The neutrophil-derived cathelicidin also induces Th17 and inhibits Th1 differentiation, while cathelicidin-deficient mice inhibit Th17. This study highlights the key role of neutrophils in regulating the T-cell fate through releasing cathelicidin (176). Emerging evidence suggests that DCs play a critical role in triggering immune suppression in response to tumor-associated antigens (177). The DCs migration is crucial for cancer immune surveillance (178). This event includes the migration of DCs into tumor sites, arresting and endocytosing cellular debris or dead tumor cells, and transporting antigens to the tumor- draining lymph nodes to induce the activation of T cells (179). The recruitment of DCs mainly depend on various chemokines including CCL4/5 and XCL1, whereas CCR7 is needed for the migration of DCs to tumor-draining lymph nodes (178). Neutrophils are major producers of CCL4/5; thus, they regulate the recruitment of DCs to the TME (180). DCs as major APCs are associated with the priming of the effector CD4^+^ and CD8^+^ T-cell response (181). DCs’ major subsets are monocytes-derived DCs (moDCs), plasmacytoid DCs (pDCs), and conventional CDs (cDCs) (177). DC subset migration plays a critical role in the immune response and tumor onset and progression (182). Migration of cDC trafficking toward lymph node parenchyma for initiating a Th2-cell-dependent immune response is mediated by the chemokines CXCR7 and CXCR8 (182). It has been reported that the subpopulation of the both mouse and human neutrophils has been found to express CXCR7 (183); hence, neutrophils regulate the migration event of cDCs to lymph node parenchyma. Moreover, the recruitment of monocyte-derived DCs (moDCs) to the lymph node is mediated by CCR2 to change their phenotype into CD11c^+^CD11b^hi^Gr-1^+^, which induces Th1 responses through IL-12p70 (184). CCR2 is overexpressed in neutrophils and plays a key role in the mobilization of neutrophils from the bone marrow to the liver and as well as the recruitment of neutrophils toward inflammatory sites (185). Thus, neutrophils regulate the migration of moDCs to lymph nodes. pDCs are key producers of type 1 interferons and play an important role in the immune response (186). Recent evidence suggests that neutrophils also regulate the activity of the pDC subset by releasing NETs (187). Together, these findings demonstrate the significance of the interaction between neutrophils and DCs in orchestrating T-cell responses.

### 3.4 Interaction between neutrophils and natural killer cells

NK cells possess the strong ability of detection and killing malignant or virally infected cells (188–190). In addition, they are also the first lymphocytes that exhibited the natural ability of killing tumor cells and remained unexplored compared with cytotoxic cell therapy toward tumor treatment (191). Clinically, the abundance of NK cells have shown good prognosis in various types of solid tumors (192). Moreover, accumulating evidence has suggested that the increased density of NK cells improves the efficacy of immune checkpoint blockade therapy (193, 194). However, various studies have reported that the function of NK cells is sternly spoiled in patients with cancer and chronic diseases (195, 196). Based on the findings that neutrophils and NK cells are found in the same region of lymph nodes and the spleen, they can make conjugates (197), and neutrophils provide assistance during the intermediate steps of tumor invasion and metastasis through abolishing the activity of NK cells (198), it is believed that neutrophils are key regulators of NK cells. In addition, neutrophils have been found in orchestrating the immune response by attracting NK cells at infection sites and activating them, which, in turn, induce adaptive immune responses through triggering dendritic cell maturation (199). It has been reported that neutrophils cleaved the NK-activating receptor (NKp46) on NK cells by producing serine protease CG and resulted in the loss of the antitumor immunity of NK cells (200). Recently, Sun et al. have investigated the underlying molecular mechanism of neutrophils in the modulation of NK cell immunity. They found that neutrophils reduce the infiltration ability of NK cells in tumor-bearing mice by downregulating CCR1. In addition, their findings show that neutrophils impair the NK cells’ antitumor immunity toward lymphoma and colon cancer cells by impairing NK-activating receptor (NKp46 and NKG2D) responsiveness. The G-CSF led to increased expression of PD-L1 on neutrophils, while IL-8 led to an enhanced expression of PD-1 on NK cells, impelling the inhibition of NK cell immunity *via* the PD-L1/PD-1 axis (201).

Given the significance of NETs in regulating the tumor immune microenvironment, Teijeira et al. (93) have investigated the interaction between NETs and immune cell population, particularly NK cells. They found that the NET-mediated encapsulation of tumor cells can shield tumor cells from NK cell–mediated cytotoxicity through impeding the interaction between surrounding target cells and immune cells. Tumor cells shielded from NK cell–mediated cytotoxicity trigger tumor metastasis in mice. In addition, authors found that the NET-mediated protective coat around tumor cells against NK cells was lost, following the removal of NETs by using the DNase-I treatment. These novel findings not only unmask the critical role of neutrophils in regulating the antitumor immunity of NK cells but also attract the attention of researchers to explore the NET-mediated shielding of tumor cells and mechanistic link between NETs’ and NK cells’ antitumor immunity. To this end, the development of potent preclinical models to capture how neutrophils physically interact with NK cells in response to immune checkpoint blockade therapy can be a new avenue for designing more effective immunotherapies. In addition, a deep understanding of how neutrophils regulate NK cells’ antitumor activity in different solid tumors can provide a new roadmap for developing immunotherapies to manipulate neutrophil–NK cell communication.

## 4 Neutrophils in therapy resistance

Accumulating evidence suggests that the aberrant regulation of tumor suppressor genes or oncogenes regulates response to immune checkpoint inhibitors by engaging neutrophils. In lung cancer, the deletion of tumor suppressor STK11/LKB1 enhances the recruitment of tumor-promoting neutrophils and resistance to immune checkpoint blockade therapy (202, 203). Moreover, the activation of c-MET increases the recruitment of reactive neutrophils from the bone marrow to the lymph node and tumor tissues and results in the inhibition of T-cell expansion and function, while the inhibition of c-MET-dependent reactive neutrophil responses facilitates T-cell infiltrations into tumors and increases the efficacy of immunotherapy (204). Previous studies have reported that CXCL5 is engaged in neutrophil recruitment during inflammation (205) and drives neutrophil infiltrations into many types of tumor tissues (206, 207). A more recent study has shown that CXCL5-mediated neutrophil accumulation in lung tumor tissue suppresses the differentiation of CD8^+^ T cells and promotes resistance to immune checkpoint inhibitors, whereas the blockade of neutrophil infiltration in lung overcomes resistance against immune checkpoint blockade therapy (53). Over the years, tumor treatment has been evolved increasingly, and an overall survival rate of tumor patients has also been improved due to ever-evolving treatment options. Among diverse tumor treatment approaches, immunotherapy has emerged as a promising therapeutic approach (208).. Targeted immunotherapies to inhibit immune checkpoints PD-1/PD-L1 or cytotoxic T lymphocyte antigen 4 for restoring exhausted CD8^+^ T-cell activity or inducing CD4^+^ T lymphocyte expansion hold promise in human cancer treatment; however, a limited number of tumor patients have received clinical benefits mainly due to acquired therapeutic resistance (209–212). Therefore, there is a dire need to explore the resistance mechanisms to immune checkpoint inhibitors to improve clinical benefits. Increasing evidence suggests that the existence of the immunosuppressive neutrophils hampers the immune system from effectively killing cancer cells, creating a major barrier for thriving tumor treatment, particularly immunotherapy (22, 213, 214). In colorectal tumor patients, CD177^+^ neutrophil infiltrations were associated with dismal outcomes in patients who received antiangiogenic bevacizumab treatment (215). In triple- negative breast cancer, neutrophils showed immunosuppressive properties, thereby rendering tumor resistant to immune checkpoint inhibitors (216).

Polymorphonuclear myeloid–derived suppressor cells (PMN-MDSCs) are a type of immature low-density neutrophils (LDNs) and exhibit numerous morphological and phenotypic features of neutrophils (217, 218). It is very difficult to distinguish neutrophils from PMN-MDSCs in the same mouse, owing to the non-existence of appropriate phenotypical markers. Therefore, researchers have compared cells expressing neutrophils/PMN-MDSC-associated markers in tumor-free or tumor-bearing mice. Previously, microarray analysis–based studies have reported that PMN-MDSCs exhibit discrete transcriptomic programs compared with neutrophils. Particularly, neutrophils demonstrated an increased expression of genes associated with NF-κB signaling through IL-1, IL-6, CD-40, TLR, and TNF pathways as well as through lymphotoxin-β receptor signaling, while PMN-MDSCs demonstrated an increased expression of genes linked with the The cAMP-response element bindingprotein (CREB) pathway, G protein signaling, autophagy, and cell cycle regulation. In addition, researchers have also compared naïve neutrophils from the bone marrow with PMN-MDSCs from tumors or the spleen (219). PMN-MDSCs revealed a higher production of pro-inflammatory cytokines as well as the activation of several downstream targets of NF-κB signaling. This may explain the difference between pathologically activated PMN-MDSCs and normal bone marrow neutrophils. In the second study, classically activated neutrophils were compared with PMN-MDSCs, where classically activated neutrophils demonstrated increased levels of TNF, IL-6, and NF-κB signaling compared with PMN-MDSCs (220). Based on cancer patients and healthy donors, Veglia et al. have identified PMN-MDSCs as CD11b^high^CD15^high^CD66b^high^CD33^high^Arg1^high^S100A9^high^Lox1^high^ and classical activated neutrophils as CD11b^+^CD15^+^CD16^+^CD66b^high^Arg1^+/−^STAT3^-^S100A9^+^LOX^−^ from the peripheral blood of NSCLC patients (221). These findings are consistent with the concept that different stimuli generate both the PMN-MDSCs and classically activated neutrophils. In bladder tumor patients, increased levels of PMN-MDSCs have been detected in tumor tissues and blood mononuclear cells, and this accumulation is associated with the tumor grade and poor prognosis (222). More recently, two studies have investigated the potential role of PMN-MDSCs in immunotherapy response in immune-competent bladder tumor models. In first study, Wang et al. have compared the Bacillus Calmette–Guerin (BCG) intravesical instillation with PA-MSHA in an MB49 orthotopic bladder cancer model. The authors found that PA-MSHA exhibited greater antitumor benefits as compared to BCG, but neither treatment was found to be curative. Their findings showed that high PD-L1 expression and increased levels of PMN-MDSCs hindered the therapeutic efficacy of the treatment (223). This novel study attracts the attention of oncologists for investigating whether increased levels of PMN-MDSCs are correlated with the clinical cases of the non-muscle-invasive bladder tumor that poorly responds to BCG therapy. In the second study, Takeyama et al. have generated cisplatin-resistant bladder tumor cell lines that demonstrated an increased expression of various chemokines, including CXCL1/2 and CCL2. They found that the depletion of PMN-MDSCs decreases the growth of cisplatin-resistant tumor in mice by enhancing the infiltration of the CD8^+^ T cells; in turn, this depletion increases anti-PD-L1 immunotherapy, suggesting the promising benefit of the combinatorial treatment of anti-PD-1/PD-L1 and PMN-MDSC-incapacitating therapy in urothelial tumor patients.

## 5 Strategies to target neutrophils

Immune evasion in human tumor has been found as a multifactorial phenomenon. In recent years, this issue has attracted greater attention because resistance mechanisms are found linked with the efficacy of immune checkpoint blockade therapy. Accumulating evidences both in human and mouse models suggests that neutrophils act as immunosuppressive players in the TME. The inflammatory chemokines and cytokines engaged in altering the function of neutrophils constitute new hot topics in the clinical tumor study, both as novel immunotherapy targets and biomarkers (224). Given the stoutness to preclinical studies incriminating neutrophils in immune checkpoint blockade failures, numerous clinical trials are going on to antagonize neutrophil activity combined with immune checkpoint blockade therapy (**Table 2**). In this section, we will shed light on novel strategies to target neutrophils that include three aspects: limiting neutrophil polarization and recruitment, reducing the immunosuppressive ability of neutrophils, and inhibiting NET production.

**Table 2:** List of clinical trials investigating effect of immune checkpoint inhibitors in combination with other drugs to overcome neutrophil-associated resistance in different cancers.

Immune checkpoint inhibitors	Drug	Target	Tumor type	Phase	Trial ID
**Pembrolizumab**	SX-682	CXCR1/2	Melanoma	I	NCT03161431
**Nivolumab**	SX-682	CXCR1/2	Colorectal cancer	Ib/II	NCT04599140
**Nivolumab**	INCB001158	Arginase-1	Solid tumors	I/II	NCT02903914
**Durvalumab**	IPH5401	C5aR	Solid tumors	I	NCT03665129
**Nivolumab**	BMS-86253	IL-8	Hepatocellular carcinoma	II	NCT04050462
**Niolumab**	BMS-13160	CCR2/5	Colorectal and Pancreatic tumors	I/II	NCT03184870
**Niolumab**	Galunisertib	TGF-β	Solid tumors	Ib/II	NCT02734160

### 5.1 Limiting neutrophil polarization and recruitment

**Figure 3:**
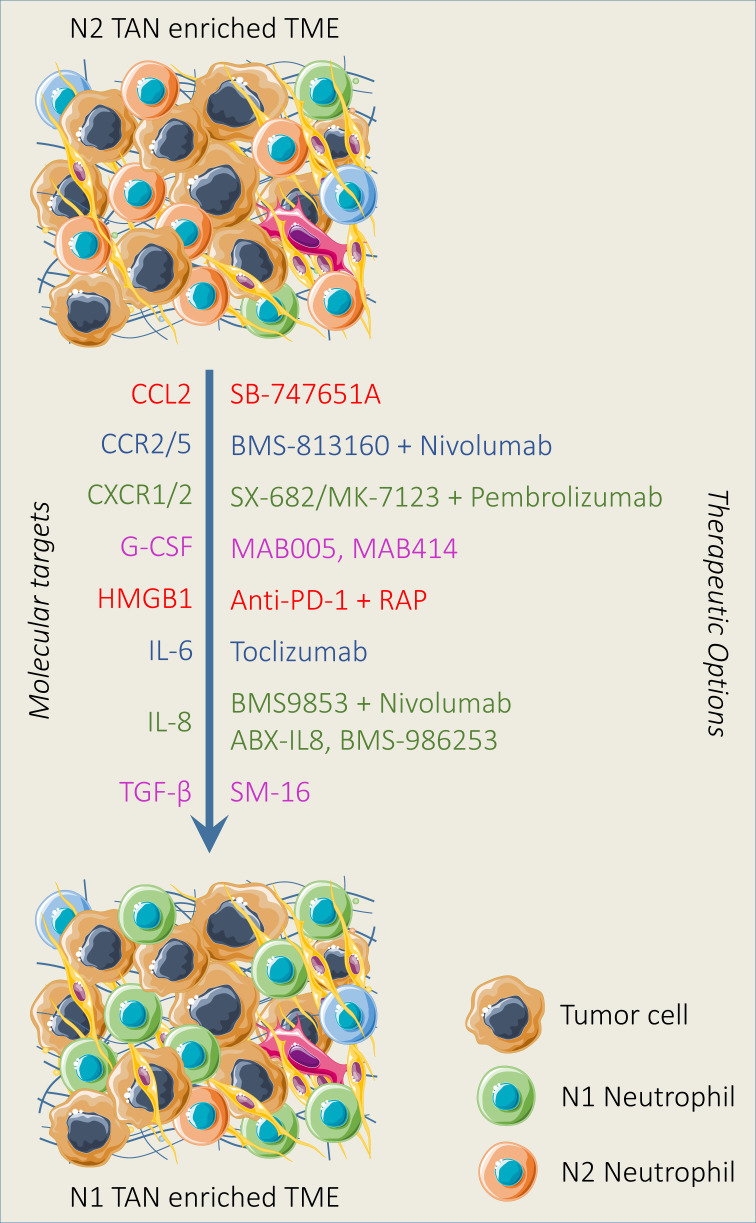
Therapeutic strategies reducing neutrophil recruitment and polarization. Various neutrophil-targeting approaches have been established and demonstrated promising outcomes both in preclinical and clinical settings. Neutrophil recruitment and polarization can be limited by the pharmacological blockade of mediators and downstream pathways alone or in combination with immunotherapy involved in neutrophil recruitment and polarization. In addition, N2 TAN can be reprogrammed into N1 TAN by modulating the activity of TGF-β and IFN-β in the TME.

In this section, we highlight the targeting of various signaling pathways that promote tumor and hinder immunotherapy efficacy through regulating neutrophil polarization and recruitment (**Figure 3**).

#### 5.1.1 Transforming growth factor beta blockade

Tumor-associated neutrophils (TANs) are polarized from an antitumor (N1) to a protumor (N2) phenotype after infiltration into the TME (225). As a major immunosuppressive cytokine, TGF-β regulates this phenotypic switch of TANs in the TME, which, in turn, suppresses the antitumor activity of NK cells and T cells (226). It has been suggested that the blockade of the TGF-β pathway suppresses colorectal cancer progression through reversing TAN polarization into the N2 type (227). In NSCLCs, the blockade of TGF-β also inhibits tumor growth through TAN polarization toward antitumor phenotypes (228). Furthermore, the TGF-β pathway plays a critical role in the recruitment of neutrophils to tumor and subsequent resistance of cancer to immune checkpoint inhibitors (52, 210, 229, 230). In recent years, the blockade of TGF-β has emerged as promising approach to induce CD8^+^ T-cell infiltration and improve immunotherapy efficacy. Martin et al. has recently investigated the potential impact of TGF-β inhibition for modulating immune checkpoint resistance in mice. Results show that the combined treatment of the anti-PD-1 antibody and SRK-181-mIG1 contributes to antitumor responses and survival benefits. In addition, this novel combinatorial strategy has resulted in the reduction of immunosuppressive myeloid cells and induction of intratumoral CD8^+^ T cells, thereby suggesting that the inhibition of TGF-β can modulate resistance to immune checkpoints (231). Previously, it has been reported that the TGF-β pathway stimulates T-cell exclusion and lymphocyte confiscation at tumor outskirts, thereby leading to tumor metastasis and resistance toward T-cell-mediated immune therapies (232, 233). The repression of CXCR3 activation has been suggested as a new mechanism for T-cell exclusion to tumors mediated by TGF-β signaling. This shows extensive applicability for tuning the efficacy of the CD8^+^ T -cell-mediated immunotherapies that entailed the infiltrations of the T cells into tumors (234). For instance, evidence suggests that TGF-β is associated with resistance toward immune checkpoint blockade (235); this may be, in part, due to diminished trafficking to tumor *via* CXCR3 repression. These findings provide a mechanistic rationale for synergy between TGF-β suppression and immune checkpoint blockade (236, 237). Furthermore, clinical trials are also being conducted to evaluate the combined treatment of galunisertib and immune checkpoint inhibitors to target TGF-β in the treatment of solid tumors (NCT02734160). Thus, the combined repression of the TGF-β pathway and immune checkpoints can be a promising avenue to improve the efficacy of forthcoming immunotherapeutic agents.

#### 5.1.2 CXCL8 blockade

CXCL8 (IL-8) has been proposed as a key player of neutrophil recruiter and also an important driver of neutrophil activity. Growing evidence has revealed the crucial role of CXCL8-CXCR1/2 axis in the TME and prognostic significance of the CXCL8 serum level in human cancer, following immune checkpoint blockade therapy (58, 238–240). Advanced NSCLC patients who responded well toward nivolumab, a PD-1 targeted treatment, demonstrated low levels of CXCL8, TNF-α, and IP-10 and high levels of BMP-9 compared to non-responders (241). Recently, two clinical studies published in Nature Medicine have shown that the neutrophil-attractant IL-8 acts as an undesirable predictive factor in tumor patients who received immune checkpoint blockade therapy. Results showed that the elevated IL-8 serum level at baseline was associated with dwindled overall survival and partial response to immune checkpoint inhibitors because of neutrophil-mediated immune suppression (242, 243). Furthermore, the increased CXCL8 serum level is linked with neutrophil expansion and limited clinical outcome from immune checkpoint blockade therapies (244, 245). In ovarian tumor, CXCL8 promotes the recruitment of tumor associated neutrophils in TME and activates JAG2 in tumor-associated neutrophils, which, in turn, suppresses the activity of the CD8^+^ T cells (129). It has been reported that the anti-CXCL8 monoclonal antibody abolishes the recruitment of neutrophils into tumor and increases the antitumor immunity of triple- negative breast cancer (246). Moreover, various clinical trials of combining the anti-PD1 antibody, nivolumab, and CXCL8 antibodies have also been carried out in both advanced solid tumors (NCT03184870), hepatocellular carcinoma (NCT04050462), and NSCLC (NCT0413379). In triple-negative breast cancer, the direct targeting of CXCL8 by using the monoclonal antibody HuMax-IL8 significantly reduces PMN-MDSC infiltration to the TME and improves immunotherapy efficacy (246). Giving importance to the neutrophil-induced M2-like macrophage phenotype, the blocking of the neutrophil accumulation in the TME represents a promising therapeutic approach to hinder neutrophil-mediated immune suppression. In this line, the latest study has shown that the blocking of CXCL-8 signaling suppresses neutrophil migration and the neutrophil-mediated polarization of anti-inflammatory macrophages in the TME (247). The above discussed findings support that CXCL8 plasma levels can provide a glimpse into the immunosuppressive TME and patients may benefit from immune checkpoint blockade therapy, following the reduction of the CXCL8 level.

#### 5.1.3 Blocking of CXCR1/2 axis

CXCR1 and CXCR2 are major chemokine receptors expressed by neutrophils (248, 249). Both the CXCR1 and CXCR2 chemokine receptors are prognostic biomarkers in many types of human cancers (250–253). The targeting of CXCR1/2 reduces the neutrophil population in several cancer models and also inhibits tumor growth and metastasis (254, 255). In addition, the inhibition of CXCR1/2 by ladarixin decreases neutrophil-dependent airway inflammation in mice (256). SX-682 is another novel potent inhibitor of CXCR1/2 (22, 71) and recently being investigated in many clinical trials in several cancer types such as melanoma, colorectal cancer, and breast cancer (NCT03161431, NCT04574583, and NCT04245397). In lung and breast cancer, the combined inhibition of CXCR1/2 by using the SX-682 inhibitor and anti-PD-1/PD-L1 has effectively controlled tumor growth in a murine model. Grippingly, this combinatorial approach demonstrated enhanced efficacy due to the decreased infiltration of granulocytic MDSC and increased population of activated T cells at the tumor site (257). Moreover, the disruption of CXCR1/2 through SX-682 has shown efficacy in mice (21, 258), and its combinatorial treatment with PD-L1-targeted antibody, pembrolizumab, is also being studied in a phase 1 trial in melanoma (NCT03161421). The mutated Kirsten rat sarcoma virus (KRAS) contributes to resistance against immune checkpoint inhibitors through recruiting PMN-MDSCs in a colorectal cancer mouse model. Mechanistically, oncogenic KRAS mutation inhibits IRF2, thereby leading to the activation of CXCL3, a potent ligand of CXCR2. The blocking of CXCR2 through CXCL3 knockdown or SX-682 treatment in mice reduces PMN-MDSC recruitment and enhances response to anti-PD-1 therapy (71). Therefore, employing an alike rationale in the preclinical models of different cancer types, SX-682 treatment demonstrated promising outcomes in decreasing the infiltration of PMN-MDSCs and synergy with several modes of immunotherapy, including adoptive T-cell transfer, immune checkpoint inhibitors, and NK cell–based therapy (259, 260). In triple-negative breast tumor patients, the phase I clinical trial (NCT02001974) to evaluate the safety of the oral administration of CXCR1/2 inhibitor reparixin has been conducted combined with paclitaxel (261). In addition, the phase II clinical trial (NCT02370238) has also been conducted to investigate the survival of triple-negative breast tumor patients, following the combined treatment of reparixin and paclitaxel.

#### 5.1.4 Blocking IL-17

IL-17 is a prominent cytokine that plays a critical role in tumor progression and immune response (262–265). It is linked with poor prognosis and drives resistance in solid tumors (266, 267) and also acts as a prognostic biomarker in different types of human cancers (268, 269). Recently, Wu et al. have investigated the potential role of IL-17 in regulating breast cancer metastasis and therapy resistance. Results show that IL-17 contributes to breast tumor metastasis and therapy resistance through recruiting neutrophils to the TME (270). Another recent study has shown that IL-17 contributes to immune suppression immunotherapy resistance by increasing the neutrophil population and NET production in the TME. However, the blocking of IL-17 enhances the sensitivity of PD-1 and CTLA4 in pancreatic cancer (55). G-CSF plays a critical role in neutrophil production and recruitment (271, 272). The targeting of the IL-23-IL-17 axis has been found to reduce neutrophil abundance induced by G-CSF (273, 274). Thus, G-CSF inhibition leads to reduce the neutrophil amount and improve antitumor efficacy in several preclinical cancer models (275).

### 5.2 Reducing immunosuppressive ability of neutrophils

Immunosuppressive neutrophils obstruct the antitumor activity of the immune system and pose a major obstacle in tumor eradication, particularly immunotherapy (276). A deep understanding of various signaling pathways regulating neutrophil activity in tumor progression have enlightened several strategies that exploit antibodies or drugs to block TANs (277, 278). Therefore, the targeting of key pathways that promote immune suppression and regulate neutrophils’ immunosuppressive function can help in reducing the immunosuppressive ability of neutrophils.

#### 5.2.1 ARG1 blockade

ARG1 is an immunosuppressive marker induced almost solely by polymorphonuclear granulocytes (PMNs) in human and regulates both the innate and adaptive immunity (279, 280). ARG1 expression is linked with increased tumor growth and immune suppression (280, 281) and serves as a prognostic biomarker in a wide range of human cancers (282–284). The presence of ARG1 in the TME suppresses both the expression of T-cell receptors and T-cell proliferation, while the inhibition of ARG1 prevents the PMN-mediated suppression of T cells (285). In several xenograft models, ARG1 inhibition has shown delayed tumor growth and enhanced PD-L1 blockade response (286). Based on the above studies, recently, various ARG1 inhibitors, including CB-1158 and OATD-02, have been selected for clinical trials in tumor immunotherapy (287). The CB-1158 potent inhibitor has been found to effectively inhibit human ARG1 (288). Furthermore, clinical trials are also being conducted to evaluate the combined treatment of CB-1158 and immune checkpoint inhibitors in the treatment of solid tumors (NCT02903914, NCT03361228, and NCT03314935). In epithelial ovarian cancer, increased ARG1 expression has been found to contribute in tumor growth and immune suppression, while the blocking of ARG1 mitigated ARG1-mediated tumor progression and immune response (289). OATD-02 inhibitor has been also entered into phase I trials, but this inhibitor has demonstrated low clearance and modest oral bioavailability (290). Importantly, a recent study has evaluated the antitumor effect of the combinatorial treatment of another potent inhibitor of ARG1 (OAT-1746) and anti-PD-1 therapy in a glioma murine model. Results showed that the combined treatment of OAT-1746 and anti-PD-1 antibody reduced tumor growth by decreasing levels of CCL2 and CCL5 in the blood plasma of mouse (291).

#### 5.2.2 Blocking C3A/C5A

The complement system is a major arm of innate immunity (292). C3A and C5A are key components of complement system and regulate immune response and tumor growth in wide range of solid tumors (59, 293–296). In addition, both the C3A and C5A promote resistance to immune checkpoints blockade therapy (283, 284). The C3A and C5A trigger inflammatory response which is crucial step in tumor onset and progression by activating leukocytes, releasing histamine, and stimulating generation of inflammatory mediators such as IL-1, IL-6, IL-1β, IFNγ, and TNF-α (293, 297, 298). A growing body of evidence suggests that C3A and C5A as key components of complement system stimulate neutrophils activation and migration (299–302). Various studies have reported that targeting of C3A and C5A and their receptors is a novel strategy for increasing immunotherapy efficacy (303, 304). Ajona et al. have investigated antitumor synergistic effect of combinatorial inhibition of C5A and PD-1 in the treatment of lung cancer. In this study, they used RMP1-14 antibody to inhibit PD-1 and L-aptamer to block C5A. Authors found that blocking of C5A downregulates immune suppression induced by MDCs as C5A promotes lung cancer onset and progression through inducing the immunosuppressive TME where MDCs are implicated. By using various lung cancer models, the authors found that the combined targeting of C5a and PD-1 significantly suppresses tumor growth and also enhances overall survival rate (305). Moreover, targeting of C5aR increases paclitaxel response in squamous cell carcinoma by reprogramming the immunosuppressive tumor immune microenvironment, thereby leading to improved CD8^+^ T cell-mediated antitumor immune response (306). In addition to this, the inhibition of complement receptors such as C3aR and C5aR has been proven to be very effective in enhancing the efficacy of immunotherapy (307). Recently, a phase I clinical trial (STELLAR-001) has also been conducted by Innate Pharma to investigate the therapeutic effect of IPH5401 (anti-C5aR) combined with durvalumab (anti-PD-1) in advanced solid tumors (308).

### 5.3 Targeting of neutrophil extracellular traps

**Figure 4:**
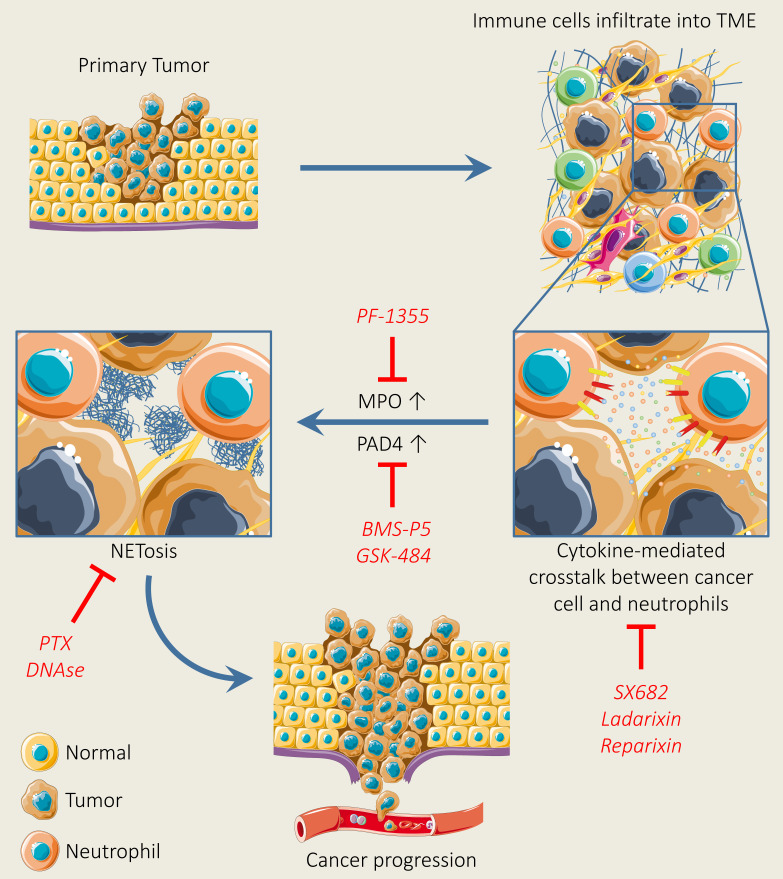
Neutrophil extracellular trap (NET) formation and inhibition for tumor therapy. NET formation is initiated, following the activation of chemokine receptors upon the secretion of various types of chemokines and cytokines secreted by tumor cells. Upon the initiation of NET formation, MPO facilitates cell cytoskeleton degradation, whereas PAD4 triggers histone citrullination, thereby leading to NET release. However, the pharmacological inhibition of chemokines, cytokines, and receptors, MPO, and PAD4 by using selective inhibitors can suppress NET formation. In addition, NET formation can be abolished by using pertussis-toxin and DNase I.

Recently, it has been confirmed that NETs do not only regulate the immune system but are also involved in the pathogenesis of various inflammatory diseases and multiple tumors (309–311). At the initial stage of tumor, NETosis favors EMT induction. The treatment of breast tumor cells with NETs drive the mesenchymal phenotype, leading to tumor progression (312). Moreover, various preclinical studies have also reported that neutrophil accumulation drives metastatic disease through hindering antitumor immune responses, by supporting cancer cell migration and producing neutrophil extracellular traps (NETs) (100, 140, 313, 314). Thus, the targeting of NETs could be a promising strategy for tumor therapy. Various strategies can be adopted for the targeting of NETs such as the blocking of pathways involved in NET production, destroying NET structure, and obstructing NET–tumor interaction (315). CXCL8 has been found to support the production of NETs (316). In addition, Yang et al. have reported that CXCL8 establishes a positive loop between NETs and colorectal cancer liver metastasis (317). Another study has shown that CXCL8 induces the production of NETs through communicating with CXCR2, which, in turn, promotes cancer cell proliferation and migration (318). Therefore, the targeting of the CXCL8-CXCR1/2 axis can be a promising approach for increasing the efficacy of immune checkpoint inhibitors. More recently, Kaiser et al. have investigated the profound impact of CXCL8 blockade in neutrophil activation and NET production. Results show that the targeting of CXCL8-CXCR1/2 by using the anti-IL-8 antibody or a clinically available CXCR1/2 blocker (reparixin) reduces neutrophil activation and NET production in mice (319). Peptidyl arginine deiminase 4 (PAD4) is a critical enzyme associated with the formation of NETs. The blocking of PAD4 reduces NETs protumor effects in various disease models (320, 321). To date, several compounds have been reported for inhibiting PAD activity such as Cl-amidine and BMS-P5. These novel inhibitors effectively abrogate NET formation induced by tumor cells and also set back disease progression (322, 323). In addition, the antitumor drug kaempferol inhibits NET formation by suppressing ROS-PAD4 signaling (324). Another novel PAD4 inhibitor (GSK484) and A_2A_ receptor (CGS21680) effectively block NET formation (325). A more recent study has shown that an Food and Drug Administration (FDA)-approved drug (disulfiram) blocks NET formation and reduces lung injury in rodents (326). Another promising strategy to inhibit NET formation is to demolish the NET structure by using (327). DNase destroys the NET backbone and results in NET degradation (328). In addition, DNase treatment has shown reduced tumor burden in a breast tumor mouse model (329). Coated nanocarriers have demonstrated higher tumor-inhibiting potential due to DNase stability linked with nanocarriers in blood. Dnase nanocarriers effectively digested NETs and suppressed breast tumor lung metastasis configuration (100) (**Figure 4**). The above findings highlight teamwork between tumor cells, TANs, and the formation of NETs in the TME and the crucial role of NETs in tumor progression and metastasis. In addition, these findings suggest that the combinatorial approach of NET blocking with immunotherapy may move toward clinic.

### 5.4 Potential role of neutrophil-derived extracellular vesicles in tumor

Activated neutrophils have been found to produce a greater amount of extracellular vesicles (EVs) than tumor cells (330). Neutrophils produce EVs either instinctively or in response to different stimuli such as chemokines, cytokines, antibodies, complement component, and bacterial stimulation (331, 332). Neutrophils exploit diverse mechanisms either to promote or suppress tumor. Among them, one key mechanism involves the release of EVs, which induce specific signaling pathways in different target cells and immune cells (333). EVs released from neutrophils exert either a pro-inflammatory or anti-inflammatory effect, mainly depending on environmental conditions (334). Neutrophils hold natural potential to traverse the blood–brain barrier (335) and rapidly penetrate into the glioma tumor site (336). Therefore, in recent years, neutrophil-derived EVs have been studied as drug delivery vehicles in tumor therapy (337). Previously, Wang et al. have shown that neutrophil–exosome-loaded drugs effectively penetrate the blood–brain barrier and migrate into brain. In addition, the intravenous injection of the neutrophil–exosome-loaded PDX efficiently inhibits tumor growth in a glioma mouse model (338). Similarly, neutrophil-carrying PDX have also been found effective in overcoming the blood–brain barrier and suppress glioma recurrence, following tumor surgery (339). More recently, Zhang et al. have engineered neutrophil-derived exosome-like vesicles and investigated the potential of this novel nanocarrier for safe drug delivery into tumor. Results show that this nanocarrier efficiently accumulates at the targeted tumor site under an external magnetic field, suppresses tumor growth, and increases the survival rate in tumor-bearing mice (340).

## 6 Unsolved mysteries and future perspectives

Over the years, the efficacy of immune checkpoint blockade therapies is obstructed in many cancer settings, which raises new unexplored scientific questions. Meantime, neutrophils have emerged as quintessential warriors of the immune system that play a key role in modulating immunotherapy efficacy; therefore, deep understanding is required for developing effective neutrophil-targeting approaches for tumor therapy. Regarding the mechanistic perspective, we highlight the following unsolved questions that, when answered, may aid in developing effective therapeutic windows to increase immunotherapy efficacy.

(a) What kind of molecular machinery controls the production of the neutrophils recruiting arbitrators in tumor cells?

(b) What kind of trafficking molecules control a precise coordination between chemokines and their receptors in the formation of NETs?

(c) Which trafficking molecules control the transcriptional programming of neutrophils in acquiring their tissue-associated features?

The neutrophil function in tumors is profoundly dictated by a precise TME. The precise TME is regulated by TGF-β and IFN-β. Therefore, modulating the activity of TGF-β and IFN-β in the TME can change the neutrophil phenotype and may unlock the therapeutic potential of the TME in dictating neutrophils as either a tumor promoter or suppressor. Thus, getting control on the desired TME modulation might be a novel approach to improve immunotherapy efficacy. Although this aspiration seems baffling, it can be accomplished by accelerating translational research. Immune checkpoint blockade therapy mainly works by dictating T cells to unleash their potential in killing cancer cells. However, immune-suppressive PMN was found to work as gatekeeper to protect cancer cells from a T-cell attack, which promotes resistance to immune checkpoint blockade therapy (341). Therefore, the identification of novel biomarkers to differentiate immunosuppressive and normal PMN by using high-dimensional mass cytometry and single-cell sequencing technology will be useful for a selective targeting of immunosuppressive PMN. In addition, the identification of novel molecules and pathways involved in the regulation of immunosuppressive PMN will also be helpful for a selective targeting of the immunosuppressive PMN. A better understanding of neutrophil ontogeny is also critical to differentiate different types of neutrophil progenitors. Neutrophils are produced from hematopoietic stem cells (HSCs) in spleen and bone marrow and then progress to common myeloid progenitors (CMP) and committed granulocyte monocyte progenitors (GMP), and then, finally, GMP yields to both neutrophils and monocytes (342). In humans, more recent studies have defined neutrophil-committed progenitors as CD66b^–^CD64^dim^CD115^–^ in SSC^low^ CD45^dim^ CD34^+^ and CD34^low/–^ (343), and early neutrophil progenitors as Lin^–^ CD66b^+/low^ CD15^low^ CD49d^+^ CD11b^–^  (344). Another more recent study has investigated the neutrophil progenitor commitment event in a human neutrophil deficiency model by using small-molecule alpha-lipoic acid. Authors have identified novel role of alpha-lipoic acid in the regulation of neutrophil lineage specification and also found that the SF3B1-ELK axis controls commitment of the human neutrophil progenitors from CD371^+^CD115^-^GMPs (345). Moreover, Zhu et al. identified committed unipotent early-stage neutrophils progenitors from the bone marrow of the human and mouse by using single-cell RNA-sequencing technology and mass cytometry. Results showed that early-stage neutrophil progenitors promote tumor both in humans and mice. Importantly, authors identified human neutrophil progenitors (hNeP) from patients’ blood with melanoma, indicating that hNeP was released from bone marrow in tumor patients and can be detected in human blood (346). As hNeP has been detected as a tumor promotor, therefore, it could be a novel immune-oncology target. Furthermore, a better understanding of the underlying molecular mechanism of the generation of the PMN-MDSCs from their progenitors is also very important in designing novel therapies to target PMN-MDSCs. Accumulating evidence suggests that the developmental joint in granulopoiesis is snuggly choreographed by various growth factors such as G-CSF, GM-CSF, and M-CSF and transcriptional factors. These master regulators play a key role during the development and maturation of the normal granulocytes; however, during tumor burden conditions, this regulatory network is dysregulated and impairs the myeloid differentiation event and drives PMN-MDSC accumulation (347, 348). We hope that this novel immuno-oncology milestone can be a motivational window for preclinical and translational scientists in developing more effective immunotherapies in this space.

## Author contributions

KZ, UR, ST, and XH wrote the manuscript. KZ, LJ, WX, and XH reviewed and edited the manuscript. KZ and UR revised the manuscript. XH supervised the project. All authors contributed to the article and approved the submitted version.

## Funding

This work was supported by NIH/NCI grants (R01 CA221158-05, R01 CA224493-04, and R01 CA240952-02 to XH). This work was also supported by the IU Simon Comprehensive Cancer Center (Grant P30CA082709).

## Conflict of interest

The authors declare that the research was conducted in the absence of any commercial or financial relationships that could be construed as a potential conflict of interest.

## Publisher’s note

All claims expressed in this article are solely those of the authors and do not necessarily represent those of their affiliated organizations, or those of the publisher, the editors and the reviewers. Any product that may be evaluated in this article, or claim that may be made by its manufacturer, is not guaranteed or endorsed by the publisher.
